# u-PA inhibitor amiloride suppresses peritoneal metastasis in gastric cancer

**DOI:** 10.1186/1477-7819-10-270

**Published:** 2012-12-12

**Authors:** Youcheng Ding, Hui Zhang, Zhuqing Zhou, Mingan Zhong, Qiliang Chen, Xujing Wang, Zhenggang Zhu

**Affiliations:** 1 Department of General Surgery, Shanghai East Hospital Affiliated to Tonggi University, 150 Jimo Rd, Pudong New Area, Shanghai 200120, China; 2 Department of Gastroenterology, Ruijin Hospital, Shanghai Jiao Tong University School of Medicine, 197 Ruijin Road II, Shanghai 200025, China

**Keywords:** u-PA inhibitor, Amiloride, Peritoneal metastasis, Gastric cancer

## Abstract

**Background:**

Peritoneal metastasis in gastric cancer represents a ubiquitous human health problem but effective therapies with limited side effects are still lacking. Although previous research suggested that u-PA was involved in some tumor metastasis such as lung-specific metastasis, the role of u-PA for peritoneal metastasis in gastric cancer is still unclear. The aim of this study was to explore whether selective pharmacological blockade of u-PA is able to affect the peritoneal metastasis of gastric cancer both *in vivo* and *in vitro*.

**Methods:**

In the present study, we evaluated the effects and explored the anti-tumor mechanisms of amiloride, a selective u-PA inhibitor, on a panel of gastric cancer cell lines and in a murine model of human gastric cancer MKN45.

**Results:**

The study showed that amiloride significantly inhibited the tumor growth and prolonged the survival of the tumor-bearing mice. In *vitro*, compared with controls, amiloride could not only significantly down-regulate the mRNA expression and protein level of u-PA from MKN45 cells with dose dependence but also inhibit the adhesion of HMrSV5 cells, migration and invasion of MKN45 cells.

**Conclusions:**

The findings in our current report provide evidence that selective u-PA inhibitor amiloride has potent effects against peritoneal metastasis in gastric cancer, suggesting its possible therapeutic value for the treatment of gastric cancer.

## Background

Metastasis and recurrence of peritoneal cancer, especially peritoneal metastasis in gastric cancer, which is often associated with lymphatic infiltration, is a prevalent cause of death in patients with gastric cancer in clinical practice [1-4]. Therapy for peritoneal metastasis in gastric cancer have been widely studied. Surgical resection is still the only effective treatment for localized disease; however, most gastric cancer patients have regional or distant metastasis at the time of their initial presentation [5]. Effective drugs with limited side effects are still lacking and the precise mechanisms are not fully understood.

Metastasis is a complex process that mediates detachment of cancer cells from a primary site, invasion into surrounding tissues, spread through the circulation, extravasion and proliferation in distant organs [6]. The urokinase (u-PA) is a pivotal proteolytic enzyme known to regulate the process of metastasis through degrading extracellular matrix (ECM). In recent years, evidence increasingly suggests that the level of u-PA secreted by cancer cells is positively correlated with the capacities of degrading ECM and invasion [7,8]. However, there is scarce systematic evidence available to clarify the effects of the u-PA system in gastric cancer with peritoneal metastasis.

Moreover, in recent years, amiloride, a selective u-PA inhibitor, has been proved to have interventional effects on gastric cancer. Antisense inhibition of u-PA could reduce the spread of human ovarian cancer in mice [9]. In this study, we investigated the effects and explored the anti-tumor mechanisms of amiloride, a selective u-PA inhibitor, on a panel of gastric cancer cell lines and in a mouse model of human gastric cancer, MKN45. These data might suggest an anti-cancer role of amiloride against gastric cancer with peritoneal metastasis, and might set the stage for a new therapy for gastric cancer.

## Methods

### Reagent and animals

Four to five week-old male BALB/c nude mice under SPF conditions were obtained from the Experimental Animal Center (approved by Shanghai East Hospital Affiliated to Tonggi University ethics committee.) and given free access to water and food. All experiments conformed to the animal care and use guidelines of the Institute’s Animal Care and Use Committee. Amiloride was obtained from the Sigma Company (***Hattiesburg***, USA) and was dissolved in saline.

### Cell lines and culture

Human gastric cancer cell lines, MKN45, and human mesothelial cell line, HMrSV5, were donated by a patient, Digestive Surgery Institute of Ruijin Hospital, Shanghai Jiaotong University. Cells were cultured in RPMI 1640 or Dulbecco’s modified Eagle’s medium (Life Technologies, Bedford, MA, USA) supplemented with heat-inactivated fetal bovine serum (FBS) (Gibco, Auckland, New Zealand), 100 units/ml penicillin, and 100 units/ml streptomycin in a humid chamber at 37°C under 5% CO_2_. Treated cells were cultured in fresh medium. The cells in our study were in logarithmic phase and their living rate measured by trypan blue was > 90%. Cultures can be maintained by the addition of fresh medium or replacement of medium. Alternatively, when cells are confluent, cultures can be established by 1:4 of MKN45 gastric cancer cells or 1:5 of HMrSV5 mesothelial cells with subsequent re-suspension.

### RNA isolation and RT-PCR

The MKN45 cells were added in the dishes at the amount of 2 × 10^6^ cells per dish. The incubation times after cell seeding with amiloride were 6 h, 12 h and 24 h. After incubation over these time periods, cells were treated with amiloride at concentrations of 0.01 mM, 0.1 mM or 1 mM, and RPMI 1640 with 10% FBS was used as a control. Total RNA was isolated from MKN45 cells using TRIzol reagent (Qiagen, Japan) according to the manufacturer’s instructions. The concentration of RNA was determined and cDNA was generated using total RNA with the Reverse Transcriptase kit (Promega, USA). RT-PCR products were visualized through 2% agarose gels containing ethidium bromide by electrophoresis. For amplification of the desired cDNA, the specific primers were used as follows: uPA: F:5′-AGA ATT CAC CAC CAT CGA GA-3′, R:5′-ATC AGC TTC ACA ACA GTC AT-3′; GAPDH: F:5′-GAA GGTGAAGGTCGGAGT C-3′, R:5′-GAA GAT GGT GAT GGG ATT TC-3′.

### Enzyme-linked immunosorbent assay (ELISA)

Total protein of cell lysates from MKN45 treated by amiloride were tested for the presence of u-PA. Concentration of u-PA was determined with commercial ELISA kits (Diagnostica international company Temecula, USA) according to the manufacturer’s instructions. The reported value is the mean ± SD of duplicate samples.

### u-PA activity

In brief, the MKN45 cells treated by amiloride were collected, washed three times with ice-cold phosphate-buffered saline (PBS) and lysed in ice-cold lysis buffer (50 mM Tris–HCl, 150 mM NaCl, 1 mM dithiothreitol (DTT), 0.5 mM EDTA, 1% nonidet P40 (NP40), 100 μg/ml PMSF, 1 μg/ml aprotinin, 2 μg/ml leupeptin 100 μM, sodium vanadate, PH 8.0) for 15 minutes at 1500 g centrifugation. Cellular proteins were extracted from the MKN45 treated by amiloride. u-PA activity was assessed with the u-PA Activity Assay Kit (Chemicon International company, Temecula,USA ) according to the manufacturer’s instructions. Results are presented as the mean ± SD of triplicate wells.

### Adhesion assays

The adhesion assays we tested in this study followed a previous description [10]. HMrSV5 cells were plated at a density of 1 × 10^4^ cells per well in 96-well multiwall plates 12 to 24 h prior to the assays. Then MKN45 cells were added in the 96-well multiwall plates at the concentration of 1 × 10^4^ cells/ml. The adhesion assay was determined by the MTT assay [11]. The incubation times, after cell seeding with amiloride were 6 h, 12 h and 24 h. After incubation for 6 h, 12 h or 24 h, cells were treated with amiloride at concentrations of 0.01 mM, 0.1 mM or 1 mM, and RPMI 1640 with 10% FBS was used as a control; three wells were included in each concentration. At every time point the absorbance of 570 nm was measured with SpectraMax M5 (Molecular Devices), using wells filled with 200 μl RPMI 1640 with 10% FBS as blanks. All experiments were performed in triplicate. The concentration- and time-dependent curves of the amiloride-treated MKN45 gastric cancer cell lines was generated as the % cell growth inhibition, using the following formula:

% Inhibition rate = (A570 of control cell - A570 of treated cells)/A570 of control cells × 100*%*.

Boyden chamber migration and invasion assays

The assay procedure to measure the *in vitro* migrating and invasive capacity of tumor cells was essentially the same as described in previous reports [12-14]. Assays were performed in modified Boyden chambers with 8-μm pore filter inserts for 24-well plates (BD Bioscience). Filters were coated with fibronectin (Calbiochem-Novabiochem). Human mesothelial HMrSV5 cells were added to the upper chamber at the amount of 2 × 10^4^ in 500 μl of serum-free medium and incubated for 12 to 24 h. For the migration assay, 2×10^4^ MKN45 cells per 250 μl were seeded on the 20% fibronectin-coated filters and incubated for 24 h. The lower chamber was filled with 300 μl of full medium. After incubation for 48 h, the cells were counted by the MTT assay [11]. After incubation for 6 h, 12 h or 24 h, cells were treated with amiloride in various concentrations (0.01 mM, 0.1 mM or 1 mM), and RPMI 1640 with 10% FBS was used as a control. Assays were performed in triplicate or quadruplicate. At every time point the absorbance of 570 nm was measured with SpectraMax M5 (Molecular Devices). For the invasion assay, 2×10^4^ MKN45 cells at the concentration of 8×10^4^ cells/ml were seeded on the 10% ice-cold Matrigel (BD Bioscience)-coated filters and incubated for 24 h. The lower chamber was filled with 300 μl of full medium. After incubation for 72 h, the number of penetrated single tumor cells and tumor cell colonies (collectively called invasion foci) was counted by the MTT assay [11]. Incubation times after cell seeding with amiloride were 6 h, 12 h and 24 h. At these time points, cells were respectively treated with amiloride at concentrations of 0.01 mM, 0.1 mM or 1 mM, and RPMI 1640 with 10% FBS was used as a control. Assays were performed in triplicate or quadruplicate. Every time point the absorbance at 570 nm was measured with SpectraMax M5 (Molecular Devices), using wells without cells as blanks. The invasion of the amiloride-treated MKN45 gastric cancer cell lines was generated as the % invasion rate, using the following formula:

Migration rate = A570 of lower/A570 of upper + A570 of lower × 100*%*.

### *In vivo* evaluation

The tumor model used in this study has been described previously [15]. Briefly, 5 × 10^6^ MKN45 cells were intraperitoneally injected into 4- to 5-week-old male BALB/c nude mice. The mice were randomly divided into two groups of 12 mice. Fourteen days after MKN45 implantation, the treatment groups received their first dose of amiloride dissolved in a saline solution. Amiloride dosage and administration schedules were based on our preliminary toxicologic and pharmacokinetic studies. Briefly, amiloride was given via oral administration to tumor-bearing mice at 50 mg/kg every day at the first three days of one week for a total time of four weeks. In parallel, the control group received the saline solution. General clinical observations of the mice, including determination of body weight and tumor growth (data not shown), were made twice weekly. The mice were sacrificed when they became moribund, and the sacrifice date was recorded to calculate the survival time.

### Statistical analysis

Statistical analysis was performed with the SPSS software system (SPSS for Windows, version 13.0; SPSS Inc, Chicago, IL). Parametric data were statistically analyzed by the Student’s *t*-test or one way analysis of variance (ANOVA) followed by post hoc tests when appropriate. Differences in non-parametric data were evaluated by the Mann–Whitney *U*-test. Survival curves were statistically analyzed using Kaplan-Meier test. Data were expressed as means ± SD. A significant difference was defined as *P* < 0.05.

## Results and discussion

### Reduction of u-PA protein and activity of u-PA by amiloride

**Figure 1:**
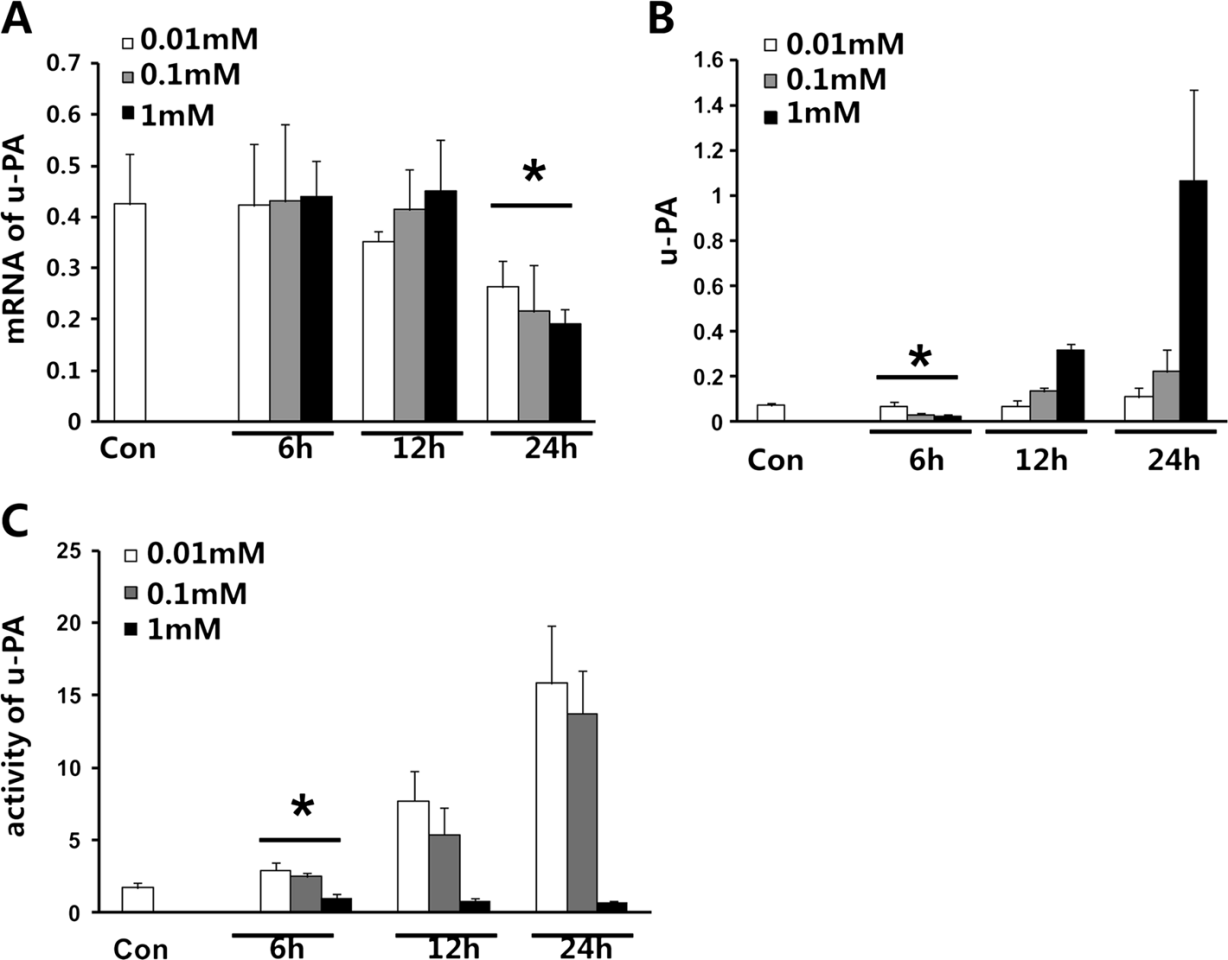
**Amiloride down**-**regulated the production of urokinase (u**-**PA) in MKN45 gastric cancer cells. **The MKN45 cells were added in the dishes at the amount of 2 × 10^6 ^cells per dish. After incubation for 6 h, 12 h or 24 h, cells were treated with amiloride in concentrations of 0.01 mM, 0.1 mM or 1 mM, and RPMI 1640 with 10% FBS was used as a control. (**A**) Expression of u-PA mRNA. (**B**) The level of u-PA. (**C**) u-PA activity. Con, control. Data are means ± SD, ^*^P < 0.05 vs. Con.

u-PA, which by its signaling is essential for the onset of gastric cancer, plays an important role in both the human and animal model of gastric cancer [16-18]. As shown in Figure 1A, the amiloride challenge led to reduction of the expressions of mRNA of u-PA after 24 h with dosage (0.01 mM to 1 mM) dependence, compared with control group (Figure 1A). The ELISA method was used to detect u-PA protein content in the cell lysates of MKN45 cells after treatment with different concentrations of amiloride. At the time point of 6 h, production of u-PA was significantly lower in the amiloride-treated group than in the control. Compared with the level of u-PA in the control (0.07 ng/ml), u-PA in the group treated with 0.1 mM to 1 mM amiloride was 0.068, 0.03 and 0.02 respectively (Figure 1B,C). However, at 12 h and 24 h, we found no significant difference between the control and amiloride group in the expression of u-PA protein. We also observed that higher dosage of amiloride could inhibit the activity of u-PA in the MHK45 gastric cancer cell line (Figure 1).

### Amiloride interfered with adhesion of mesothelial cell line HMrSV5

**Figure 2:**
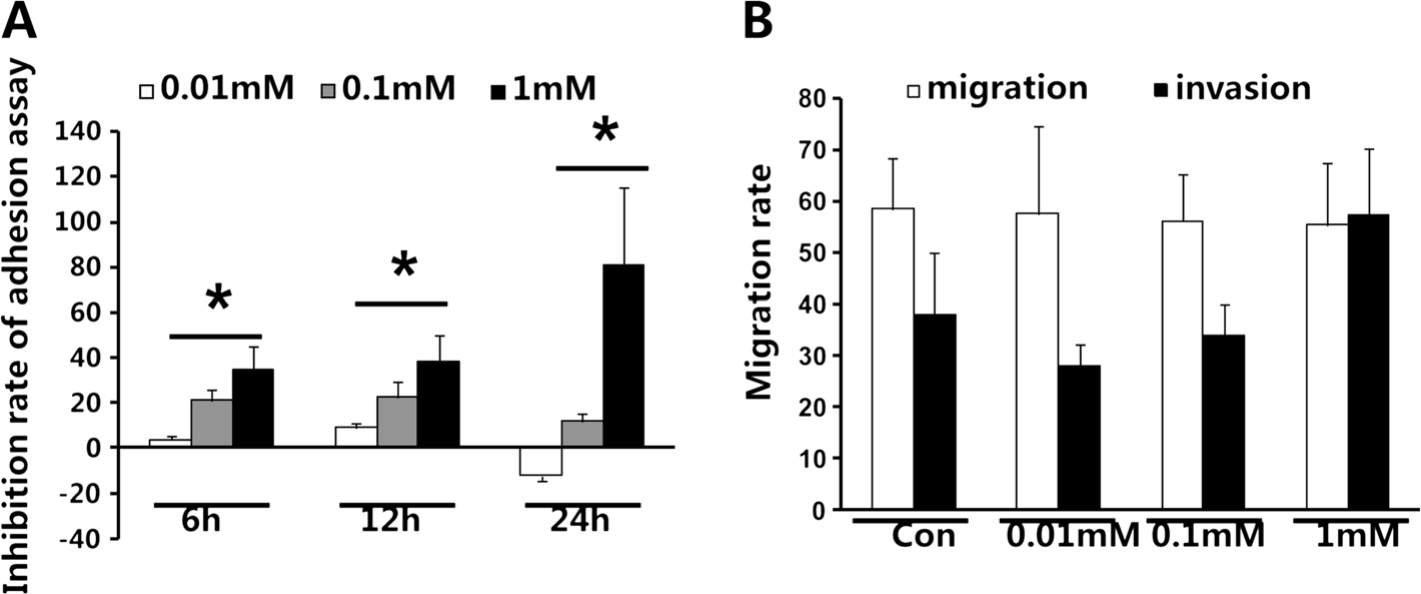
**Adhesion assay and Boyden chamber migration**/**invasion assay of amiloride treatment. **(**A**) Adhesion assay of amiloride-treated mesothelial cell line HMrSV5 tested by MTT (%). (**B**) Boyden chamber migration and invasion assay of amiloride tested by MTT (%). Con, control. Data are means ± SD, ^*^P < 0.05 vs. Con.

*In vitro*, compared with controls, amiloride significantly decreased the adhesion of mesothelial HMrSV5 cells. This inhibition was time- and dose-dependent (Figure 2A).

### Mediation of Boyden chamber migration and invasion of MKN45 gastric cancer cells *in vitro* by amiloride

We investigated the effects of amiloride on the Boyden chamber migration of MKN45 gastric cancer cells. As shown (Figure 2), amiloride slightly suppressed Boyden chamber migration of MKN45 cells. Compared with the migration rate of the control group (58.5%), the migration rates with 0.01 mM to 1 mM amiloride were 57.51%, 56.14% and 55.44%, respectively. We also found that in comparison to the invasion rate of the control group (38.04%), 0.01 mM and 0.1 mM amiloride reduced the invasion rate to 22.08% and 33.97% respectively. However, the invasion rate of 1 mM amiloride was 57.33% (Figure 2B). These results suggest that amiloride might mediate the migration and invasion of MKN45 cells (Figure 2).

### Amiloride down-regulated the mRNA expression of u-PA

**Figure 3:**
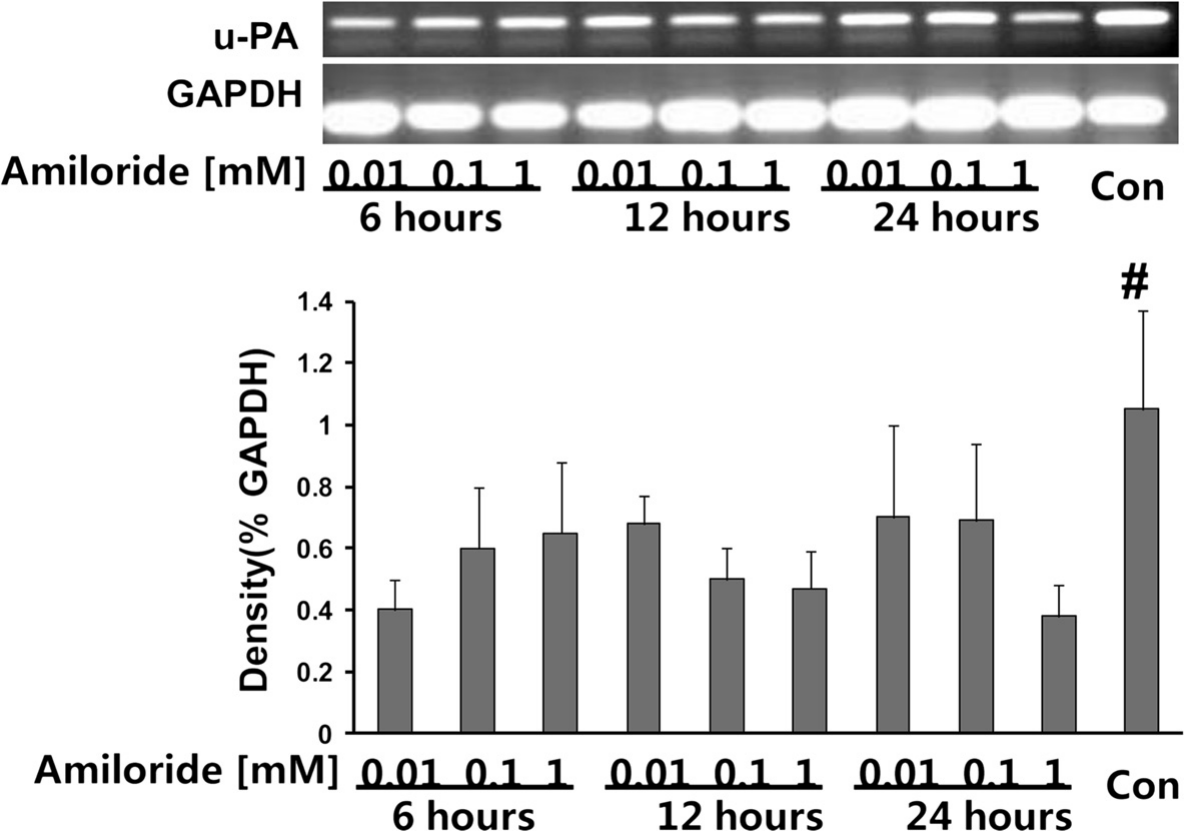
**Amiloride down**-**regulated the mRNA expression of urokinase (u**-**PA) in MKN45 gastric cancer cells. **The MKN45 cells were added at the amount of 2 × 10^6 ^cells per dish. After incubation for 6 h, 12 h or 24 h, cells were treated with amiloride in concentrations of 0.01 mM , 0.1 mM or 1 mM, and RPMI 1640 with 10% FBS was used as a control. Total RNA was isolated from MKN45 cells using TRIzol reagent (Qiagen, Japan) according to the manufacturer’s instructions. The concentration of RNA was determined and cDNA was generated using total RNA with the Reverse Transcriptase kit (Promega, USA). (**A**) RT-PCR analysis for u-PA in MKN45 gastric cancer cells. Con, control. Data are means ± SD, ^*^P < 0.02 vs. Con.

We analyzed the effects of amiloride on the mRNA expression of uPA in the gastric cancer cell line MKN45 (Figure 3). As shown, the amiloride challenge led to time- and dosage-dependent reduction of the expressions of mRNA of the u-PA from 12 h to 24 h and at 0.01 mM to 1 mM, compared with he control group (Figure 3). These results indicate that amiloride suppressed production of u-PA in MKN45 gastric cancer cells.

### Amiloride inhibited MKN45-derived tumor growth and prolonged the survival of the tumor-bearing mice

**Figure 4:**
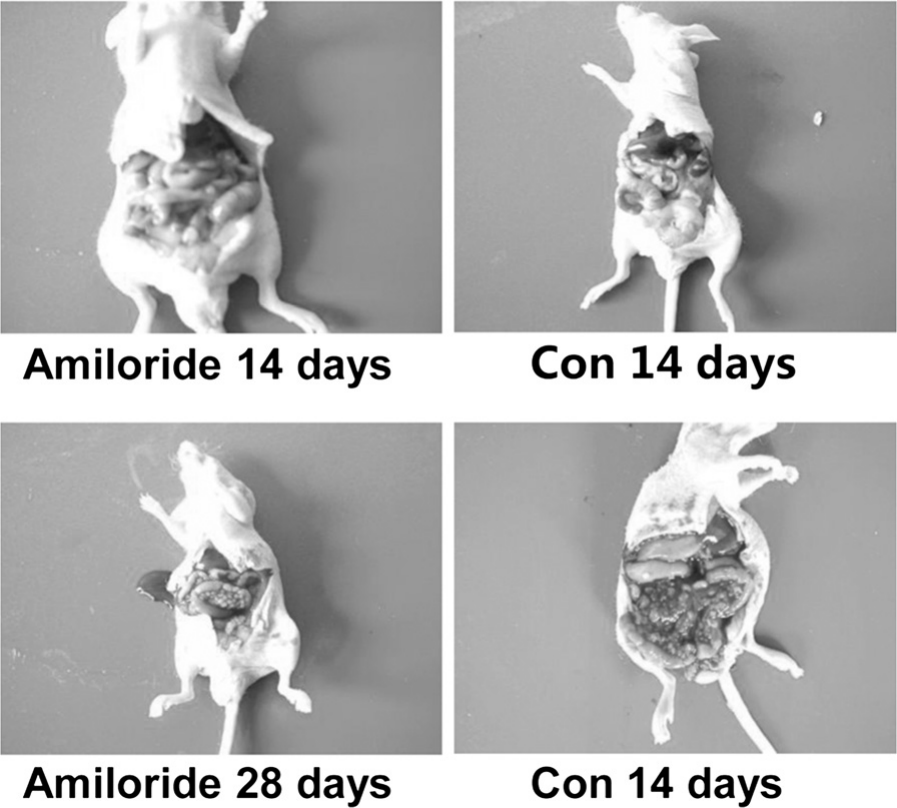
**Amiloride inhibited MKN45 derived-tumor growth. **We intraperitoneally injected 5 × 10^6 ^MKN45 cells into 4- to 5-week-old male BALB/c nude mice. The mice were randomly divided into two groups of twelve per group; 14 days after MKN45 implantation, the treatment group received the first dose of amiloride dissolved in saline solution. Dosage and administration schedules were based on our preliminary toxicologic and pharmacokinetic studies; amiloride was given orally to tumor-bearing mice at 50 mg/kg every day at the first three days of one week for a total time of four weeks. In parallel, the control group received saline solution. Mice were sacrificed when they became moribund, and the sacrifice date was recorded to calculate the survival time. (**A**) Amiloride inhibited MKN45-derived tumor growth. Con, control; Amiloride, group treated with 50 mg/kg amiloride.

**Figure 5:**
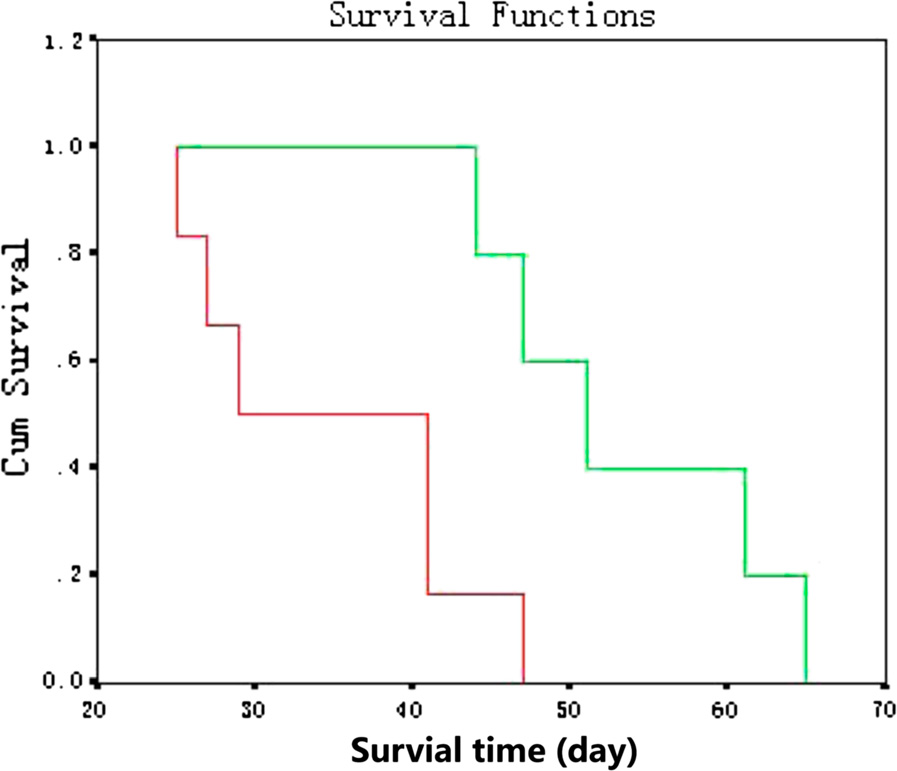
**Survival function of MKN45 gastric tumor**-**bearing mice treated with amiloride. **Briefly, 5 × 10^6 ^MKN45 cells were intraperitoneally injected into 4- to 5-week-old male BALB/c nude mice. The mice were randomly divided into two groups of six per group; 15 days after MKN45 implantation, the treatment group received the first dose of amiloride dissolved in a saline solution. Amiloride was given orally to tumor-bearing mice at 50 mg/kg every day at the first three days of one week for a total time of two weeks. In parallel, the control group received saline solution. Mice were sacrificed when they became moribund, and the sacrifice date was recorded to calculate the survival time. The red line represents control and the green line represents the amiloride group. *P*< 0.05.

Having shown that amiloride suppressed tumor cell growth, we investigated its antitumor effects in a murine model of gastric cancer. As shown in Figure 4, at the end of the experiment, the oral administration of amiloride at 50 mg/kg led to respective reductions in tumor growth, compared with that in control mice treated with saline solution. We measured the animal weight and found the weight of mice in the amiloride-treated group was decreased. We also observed appetite, fur, behavior, etcetera, to evaluate the physical status, and there were no changes in gross measures. Most intriguing, as shown in Figure 5, at the end of the study, compared with the control, the 50 mg/kg amiloride group had a 70-day survival median of 51 days. Although the 50 mg/kg group had a similar 70-day survival rate to the control, the death date was obviously delayed. These data suggest that oral administration of amiloride had effects on inhibiting MKN45-derived tumor growth and prolonging the survival of the tumor-bearing mice.

## Discussion

Treatment of human peritoneal metastasis in gastric cancer remains a major clinical problem [19]. Metastasis is associated with the process of cancer cells from a primary site invading surrounding tissues [20,21]. Here, we clearly show that blockade of the u-PA with amiloride suppresses the development of peritoneal metastasis in gastric cancer.

It is reported that amiloride, an inhibitor of Na^+^ transport, competitively inhibits the catalytic activity of u-PA, without decreasing those of t-PA, or plasmin [22]. Thus, amiloride may be a useful model compound for the development of u-PA-specific protease inhibitors [23]. u-PA is a pivotal proteolytic kinase known to regulate the process of cancer metastasis [24] . Lokman *et al*. have observed that u-PA knockdown could inhibit the proliferation of cancer cells in peritoneal metastasis [25]. Jankun *et al*. reported that treatment of amiloride could not only reduce the size of prostate cancer xenografts in severe combined immunodeficient mice, but also help survival [26]. To further study the inhibition effects of u-PA on gastric cancer peritoneal metastasis, we investigated the effects and explored the anti-tumor mechanisms of amiloride, a selective u-PA inhibitor, on a panel of gastric cancer cell lines and in a mouse model of human gastric cancer MKN45. Previous research found that amiloride participated in the transcriptional and post-transcriptional regulation of u-PA gene expression in colon cancer cells [27]. Ogura *et al*. also agreed that amiloride played a specific role in inhibiting u-PA activity [28]. We analyzed the effects of amiloride on mRNA and protein production and activity of u-PA in MKN45 gastric cancer cells *in vitro*. In accordance to previous reports, the results showed that amiloride not only decreased both mRNA and protein production of u-PA in MKN45 gastric cancer cells, but also reduced the u-PA activity of MKN45 cell line. Amiloride inhibited MKN45-derived tumor growth and prolonged the survival of the tumor-bearing mice. Consequently, it might be concluded that the inhibition of u-PA by amiloride could suppress peritoneal metastasis in gastric cancer. Furthermore, activation of Na+/H+ exchange activity is found as a ubiquitous response to early growth factors, such as u-PA [29]. uPA/uPAR-mediated tumor progression and metastasis requires Na+/H+ exchange [30]. Amiloride is reported as a Na+/H+ exchange inhibitor to inhibit cancer cell invasion and motility [31-33]. Taken together, it is believable that amiloride may first inhibit u-PA expression and then affect Na+/H+ exchange activity, ultimately resulting in suppression of peritoneal metastasis in gastric cancer.

In a recent study on breast cancer cells *in vitro*, Tuck *et al*. had found that the same as anti-u-PA antibody and anti-u-PAR antibody, amiloride significantly inhibited migration and invasion of breast cancer cells [34]. Evans *et al*. observed that oral amiloride inhibited lung metastasis in pulmonary metastasis in the rat mammary cancer model and this effect was positively correlated with time- and dose-dependence [35]. In SCID mice subcutaneously injected with prostate cancer cells, JanKun *et al*. found that oral amiloride could potently inhibit tumor growth and prolong the survival of tumor bearing mice [26]. Evans *et al*. reported that amiloride could dose- and time-dependently inhibit cancer cell metastasis [36]. In the adhesion test between MKN45 cells and mesothelial cells, we found the inhibition of adhesion by amiloride was correlated with inhibition of the growth of MKN45 cells. In addition, at the time point of 24 h, 0.01 mM amiloride had lost the inhibitive role of adhesion, which might be related to the inhibition of u-PA activity caused by long-time stimulation of amiloride at a low concentration.

## Conclusions

In our study, compared with controls, the selective u-PA inhibitor amiloride significantly decreased the adhesion of mesothelial HMrSV5 cells, and inhibited the adhesion of the gastric cancer cell MKN45 *in vitro*. However, there was no significant effect of amiloride on migration of gastric cancer cell MKN45 peritoneal metastasis. It is interesting to further elucidate the precise mechanism of cancer cell migration and invasion in gastric cancer peritoneal metastasis mediated by u-PA inhibition.

## Competing interests

The authors declare that they have no competing interests.

## Authors’ contributions

YD and HZ participated in the design of this study, and they both performed the statistical analysis. ZZ carried out the study, together with QC and MZ collected important background information, and drafted the manuscript. MZ, XW and ZZ conceived this study, participated in the design and helped to draft the manuscript. All authors read and approved the final manuscript.
